# Editorial: New methods or strategies to inactivate or control foodborne viruses

**DOI:** 10.3389/fmicb.2023.1188935

**Published:** 2023-03-30

**Authors:** Xinhui Li, Dan Li, Gloria Sánchez Moragas

**Affiliations:** ^1^Department of Microbiology, University of Wisconsin-La Crosse, La Crosse, WI, United States; ^2^Department of Food Science and Technology, National University of Singapore, Singapore, Singapore; ^3^Department of Preservation and Food Safety Technologies, Instituto de Agroquímica y Tecnología de Alimentos-Consejo Superior de Investigaciones Científicas (IATA-CSIC), Valencia, Spain

**Keywords:** foodborne viruses, noroviruses, rotavirus, inactivation, food safety

Foodborne viruses have been the leading cause of acute gastroenteritis worldwide. There is a great need for novel approaches to inactivate or control foodborne viruses as food products commonly associated with foodborne viruses are not suitable for heat treatments. Furthermore, most foodborne viruses, especially noroviruses, are resistant to alternative treatments and preservatives that can be used to control foodborne bacterial pathogens. With the latest scientific knowledge on foodborne viruses and new cultivation techniques for human noroviruses (HuNoVs), scientists around the world have been investigating and developing new controlling methods and strategies. Therefore, we launched the Research Topic in July 2021.

This volume includes four articles with research subjects of HuNoV and rotavirus. Escudero-Abarca et al. characterized the efficacy of eight commercial hand sanitizers (seven-ethanol-based and one benzalkonium chloride-based), alongside with 60% ethanol solution as a benchmark, against HuNoV with an epidemic GII.4 HuNoV strain by an *in vivo* fingerpad method, and found that product performance was variable and none of the products were able to completely inactivate the HuNoV. Using a small-scale system, Song et al. evaluated the efficacy of slightly acidic electrolyzed water (SAEW) combined with ultraviolet C-light-emitting diode (UVC-LED) in inactivating a GII.4 HuNoV strain, on a stainless-steel surface. They found that the specific treatment conditions for inactivation of the HuNoV strain were an SAEW droplet volume of 180 μL with 30 ppm available chlorine concentration and a UVC-LED exposure dose of 2 mJ/cm^2^, and suggested that the combined treatment could effectively prevent the spread of HuNoVs. Xu et al. investigated the direct interactions between lettuce-encapsulated bacteria that expressed histo-blood group antigen-like substances and a GII.4 HuNoV strain and through simulated environmental experiments, including short-time high-temperature treatment (90°C) and UV exposure, they demonstrated that binding of HuNoVs with bacteria expressing histo-blood group antigen had detrimental effects on the HuNoV reduction. Cantú-Bernal et al. examined the anti-rotavirus effect of probiotics *Bifidobacterium longum* and *Lactiplantibacillus plantarum* in combination with microalga *Chlorella sorokiniana* in a dairy product flan, and observed a potent anti-rotavirus effect of *C. sorokiniana*, as well as increased anti-rotavirus activity of the probiotics with the combination of *C. sorokiniana*.

Taken together, we hope this collection of articles provide useful and applicable information in controlling and inactivating foodborne viruses, to help prevent illness and outbreaks caused by foodborne viruses.

## Author contributions

XL: original draft preparation and editing. DL and GS: editing. All authors have approved the article.

